# Ohio State Dental Association

**Published:** 1869-02

**Authors:** 


### Proceedings of Societies.

### OHIO STATE DENTAL ASSOCIATION.

### THIRD ANNUAL MEETING.

COLUMBUS, Ohio, Dec. 1st, 1868.

Society met pursuant to adjournment. President J. Taft in the Chair. On motion, G. W. Keely was appointed Secretary *pro tem.*

Minutes of the last annual meeting, and the called meeting of May 20th, read and approved.

Dr. H. A. Smith and C. R. Butler, appointed to fill the vacancy in the Executive Committee.

Roll of members called, when the following named were present: Drs. G. W. Keely, J. Taft, H. A. Smith, C. H. Harroun, J. Williams, W. M. Herriott, M. DeCamp, F. H. Rehwinkel, T. W. French, C. R. Butler, B. Strickland, W. P. Horton, W. E. Dunn, B. T. Spellman, A. Berry, A. A. Blount, L. Buffett, N. W. Williams, D. R. Jennings, C. R. Taft, H. H. Harrison, J. L. Dunlap, W. R. Lilley, G. W. Dunn, C. M. Kelsey.

On motion, Drs. DeCamp, Rehwinkel, and H. A. Smith, were appointed to report rules by which the Committee on Membership shall be governed. This committee to report at the next annual meeting. The Committee on Membership were requested to receive applicants and report a heretofore.

The following report of the Executive Committee was adopted:

1st. Report of Officers.
2d. Election of Officers at 3 P. M.—first day.

3d. Report of Committees, and Miscellaneous business in order at any interval in discussions.

Subjects for discussion :

1st. Means for controlling the flow of saliva during operations.

2d. What are the indications for extraction of teeth.

3d. Mechanical Dentistry.

4th. Dental Medicines.

5th. Methods for the preservation of the Dental pulp.

M. DeCamp,

C. R. Butler,

H. A. Smith, } *Committee.*

The committee appointed to procure a seal, for the use of Society, and certificates for applicants, to be conferred by the Examining Board, would respectfully report : That they procured a seal at a cost of ten dollars, and certificate engraved on stone, with one hundred printed copies of same, at a cost of twenty-five dollars, which bills were paid by the Treasurer. Adopted.

Signed, H. A. Smith,

G. W. Keely,

A. Berry, } *Committee.*

Adjourned to meet at 2 P. M.

G. W. Keely, Sec. *pro. tem.*

AFTERNOON SESSION.

Minutes of morning session read and approved.

On motion, A. G. Adel, Phonographic Reporter, was employed at five dollars per session. Dr. C. R. Taft's dues for the past year were remitted, he having been out of practice during that time.

The first subject for discussion was taken up, "Means for controlling the flow of saliva during operations."

Spoken to by Drs. Spellman, Buffett, Butler, N. W. Williams, Blount, H. A. Smith and others; illuminating this subject of so much importance to the profession.

The second subject for discussion was taken up, "What are the indications for extraction of Teeth." An interesting discussion ensued, containing much information of vital importance to every person; participated in by Drs. DeCamp, Butler, Buffett, Dunlap, Kelsey, Horton, Keely, Smith, Herriott, Berry, Harroun, Spellman, I. Williams, and Rehwinkel. On motion of Dr. Keely, Dr. Hardy, of Cincinnati, was invited to participate in the discussion, who then spoke to the subject.

The hour for the election of officers for the ensuing year having arrived, a ballot was taken, when the following named were declared duly elected:

*President*—W. P. Horton, Cleveland.

1*st Vice-President*—H. A. Smith, Cincinnati.

2*d Vice-President*—F. H. Rehwinkel, Chillicothe.

*Corresponding Secretary*—C. R. Butler, Cleveland.

*Recording Secretary*—G. W. Keely, Oxford.

*Treasurer*—A. Berry, Cincinnati.

Dr. Horton, President elect was conducted to the Chair, when he made a neat little speech, and was enthusiastically applauded.

On motion of Dr. Spellman, the following resolution was passed:

*Resolved*, That a committee of three be appointed to report to this society the names of those subject to expulsion by reason of a resolution passed at the last annual session. The Chair appointed Drs. Spellman, Blount and Buffett.

Dr. Rehwinkel offered the following, which was adopted:

*Resolved*, That the Committee, composed of Dr. Spellman and others, appointed to ascertain the delinquencies of members, shall likewise investigate the condition of the books, accounts and papers of A. W. Maxwell, Secretary, and request him to turn over all funds, papers and books, now in his possession, either to the committee or the proper officer of this society.

The following report was offered and adopted. "The committee to whom was referred the matter of delinquencies of members for last year and this, would respectfully report, That the names herewith attached are already expelled, by reason of the following resolution passed at the last annual meeting of this body, viz.: *Resolved*, That any member now in debt to this society who shall continue so until the next meeting, shall appear in person or by proxy at the same, and show cause for delinquency, and those failing to do so shall cease to be members, and that the Treasurer be requested to notify delinquents."

[Seventeen names are reported, which are withheld from publication for prudential reasons.—*Sec'y.*]

Signed, B. T. SPELLMAN, A. A. BLOUNT, L. BUFFETT, } *Committee.*

In pursuance to a notice given at the last annual meeting by Dr. A. A. Blount, It was

*Resolved*, That the By-Laws be so amended as to provide that the annual meeting shall be held on the first Tuesday of December each year, and that the semi-annual meetings be discontinued.

Dr. Berry offered the following which was unanimously adopted:

*Resolved*, That the thanks of this society be tendered to Dr. C. H. James, of Cincinnati, for originating the action resulting in the passage of the Bill "To regulate the Practice of Dentistry," by the Ohio Legislature.

Adjourned to meet at 7 P. M.

EVENING SESSION.

Dr. H. A. Smith, Vice-President, in the Chair. Minutes of afternoon session read and approved.

The third subject, "Mechanical Dentistry," was then discussed with unusual interest and at length, by Drs. Berry, Spellman, Kelsey, Herriott, Dunn, and others. By permission, Dr. Kelsey made a statement of a case of fractured Lower Jaw, now under his treatment.

Dr. Keely, by request, described his treatment of a case of compound fracture of the Lower Jaw for a man seventy-six years of age; treated successfully two years since.

On motion, the society adjourned to meet Wednesday morning at 9 o'clock.

### WEDNESDAY MORNING, DEC. 2D.

Society met pursuant to adjournment. Minutes of last session read and approved.

On motion, The Executive Committee who have in charge the Rubber contest, were requested to report progress. Dr. Taft, Chairman, made a lengthy report in regard to the Cincinnati cases; this subject was further discussed with much earnestness by several members.

Committee on Membership recommended Dr. T. M. Talbott, of Galion, and Dr. Hardy, of Cincinnati, both of whom were elected.

Dr. DeCamp offered the following, which was passed. *Resolved*, That it be left to the judgment of the Executive Committee to determine whether it is best to carry up the Rubber suit, after they have consulted Col. Fisher, and after it has been determined whether a permanent injunction is maintained, and that the committee make a report to the profession, the condition of their finances.

The following was offered and adopted:

WHEREAS, The Ohio State Dental Society fully appreciates the untiring and energetic efforts made by its Committee, which had in charge the defense of the members of the Dental profession in the late Rubber suits; and whereas this Society has unlimited confidence in the sound judgment and discretion of all the gentlemen of whom this Committee is composed, be it therefore

*Resolved*, That the hearty and sincere thanks of this body be tendered to the Committee, for invaluable services rendered to the whole Dental profession.

*Resolved*, That this Society solicits the continuance of the same Committee, and confidently expresses the hope that all necessary steps will be taken by said Committee, to guard the interests of the profession in the future.

F. H. Rehwinkel.

On motion, the society adjourned to meet at 2 P. M.

AFTERNOON SESSION.

Society met. Minutes of morning session read and approved.

On motion, it was

*Resolved*, That this society pay all expenses which may accrue in making settlement with the late Secretary.

The several bills presented were referred to an Auditing Committee, composed of Drs. Herriott, Smith and Blount.

On motion of Dr. Keely, Dr. Hall's dues for the past year were remitted, on account of severe affliction.

A committee of five, composed of Drs. DeCamp, Keely, Berry, Harroun and N. W. Williams, were appointed to procure the Incorporation of this Society.

Dr. Hall, of Piqua, was called upon to give a statement of the attack made upon him by assassins, by which he came near losing his life.

Dr. Taft made a statement of his late microscopic examinations of enamel and dentine.

Dr. DeCamp, Chairman of Committee of Incorporation, reported that there was no law by which this Society could be incorporated; but that the law Regulating the Practice of Dentistry, constitutes this society a legal body. Report accepted, and committee discharged.

*Report of Auditing Committee:*

Your Committee have examined all bills presented, and find them correct, and recommend the payment in full: Bill for seal, $10; Certificates, $25. For Hall and Janitor, $14; Dr. Williams, for stationery, $2.40; Dr. Horton, for same, $7.90; Reporter, $25.

On the following, 60 per cent: F. H. Rehwinkel, $43.50;

M. DeCamp, $47; A. A. Blount, $10; J. Taft, $106; C. H. Harroun, $50; W. H. Herriott, $10.20; W. P. Horton, $54.

W. M. Herriott,<br>H. A. Smith,<br>A. A. Blount, } *Committee.*

The President announced the Standing Committees for the ensuing year:

*Executive Committee*—M. DeCamp, H. A. Smith, W. M. Herriott.

*Dental Ethics*—N. W. Williams, F. H. Rehwinkel, G. W. Keely.

*Publication*—J. Taft, G. W. Keely, B. Strickland.

*Essayists appointed*—H. A. Smith, A. Berry, C. R. Butler, J. H. Paine, C. H. Harroun.

[All of whom are expected to be prepared.—*Sec'y.*]

The Executive Committee propose the following order of busineess for the next annual meeting.

Reading minutes of last annual meeting.

Reports of Standing Committees.

Election of Officers, to be held 3 P. M., second day.

Exhibition of Instruments and appliances, from 2 to 3 P. M., second day.

SUBJECTS FOR DISCUSSION.

1st. Diseased condition of the soft tissues of the mouth, and their treatment.

2d. Disease of the antrum and treatment.

3d. Mechanical Dentistry.

4th. Necrosis of the teeth, causes and treatment.

5th. In what cases should the Dental pulp be destroyed.

M. DeCamp,<br>H. A. Smith,<br>W. M. Herriott, } *Committee.*

After attending to some miscellaneous business, the society adjourned to meet at 7 o'clock.

G. W. Keely, *Sec'y.*

EVENING SESSION.

President Horton in the Chair. Dr. Spellman was appointed to notify delinquents for dues; of the action taken by this society. Drs. DeCamp and Jennings were appointed to select delegates to attend the next annual meeting of the American Dental Association.

A recess of half an hour was allowed to examine Dental appliances. On resuming business,—

Dr. J. Taft read a very interesting paper, subject,—"The instrumentalities of professional education." This paper was approved, and discussed with much earnestness by several members, and referred to the publishing committee.

The following report was then offered and adopted:

Your committee would recommend the following names as delegates to the next meeting of the "American Dental Association;" G. W. Keely, L. Buffett, A. A. Blount, A. Berry, C. R. Butler, W. R. Lilley, B. T. Spellman, N. W. Williams, W. P. Horton.

M. DeCamp,<br>

D. R. Jennings, } *Committee.*

"Calcification of the Dental pulp" was discussed by J. Taft, Harroun, Spellman, Butler, and others.

Professor Reamy being present, made some very interesting remarks in regard to difficult dentition; several others discussed this subject.

After a late and interesting session the Society adjourned.

C. R. Butler,<br>

Secretary, *pro tem.*

